# A multicenter evaluation of viral bloodstream detections in children presenting to the Emergency Department with suspected systemic infection

**DOI:** 10.1186/s12887-021-02699-9

**Published:** 2021-05-18

**Authors:** Christina A. Rostad, Neena Kanwar, Jumi Yi, Claudia R. Morris, Jennifer Dien Bard, Amy Leber, James Dunn, Kimberle C. Chapin, Anne J. Blaschke, Judy A. Daly, Leslie A. Hueschen, Matthew Jones, Elizabeth Ott, Jeffrey Bastar, Kevin M. Bourzac, Rangaraj Selvarangan

**Affiliations:** 1 Department of Pediatrics, Emory University School of Medicine, 2015 Uppergate Drive NE, Atlanta, GA 30322 USA; 2 Children’s Healthcare of Atlanta, Atlanta, GA USA; 3 Department of Pathology and Laboratory Medicine, Children’s Mercy Hospital, Kansas City, MO USA; 4 Department of Pathology and Laboratory Medicine, Children’s Hospital Los Angeles; Keck School of Medicine, University of Southern California, CA Los Angeles, USA; 5 Nationwide Children’s Hospital, Columbus, OH USA; 6 Texas Children’s Hospital, Houston, TX USA; 7 Rhode Island Hospital, Providence, RI USA; 8 University of Utah School of Medicine, Salt Lake City, UT USA; 9 Primary Children’s Hospital, Salt Lake City, UT USA; 10 Department of Pediatrics, Children’s Mercy Hospital, University of Missouri-Kansas City School of Medicine, University of Kansas School of Medicine, Kansas City, MO USA; 11 BioFire Diagnostics, LLC, Salt Lake City, UT USA

**Keywords:** Viremia, Pediatrics, Serious bacterial infections, Enterovirus, Adenovirus, Parvovirus B19, Cytomegalovirus, Human parechovirus

## Abstract

**Background:**

Fever is a common symptom in children presenting to the Emergency Department (ED). We aimed to describe the epidemiology of systemic viral infections and their predictive values for excluding serious bacterial infections (SBIs), including bacteremia, meningitis and urinary tract infections (UTIs) in children presenting to the ED with suspected systemic infections.

**Methods:**

We enrolled children who presented to the ED with suspected systemic infections who had blood cultures obtained at seven healthcare facilities. Whole blood specimens were analyzed by an experimental multiplexed PCR test for 7 viruses. Demographic and laboratory results were abstracted.

**Results:**

Of the 1114 subjects enrolled, 245 viruses were detected in 224 (20.1%) subjects. Bacteremia, meningitis and UTI frequency in viral bloodstream-positive patients was 1.3, 0 and 10.1% compared to 2.9, 1.3 and 9.7% in viral bloodstream-negative patients respectively. Although viral bloodstream detections had a high negative predictive value for bacteremia or meningitis (NPV = 98.7%), the frequency of UTIs among these subjects remained appreciable (9/89, 10.1%) (NPV = 89.9%). Screening urinalyses were positive for leukocyte esterase in 8/9 (88.9%) of these subjects, improving the ability to distinguish UTI.

**Conclusions:**

Viral bloodstream detections were common in children presenting to the ED with suspected systemic infections. Although overall frequencies of SBIs among subjects with and without viral bloodstream detections did not differ significantly, combining whole blood viral testing with urinalysis provided high NPV for excluding SBI.

**Supplementary Information:**

The online version contains supplementary material available at 10.1186/s12887-021-02699-9.

## Background

Fever is a common symptom in children presenting to the Emergency Department (ED). Although the incidence of serious bacterial infections (SBIs) in febrile children has decreased since the implementation of routine immunizations against *Streptococcus pneumoniae* [1, 2] and *Haemophilus influenzae* type b [3], up to 7.2% of febrile children < 5 years of age have either urinary tract infection, pneumonia, or occult bacteremia [4]. Distinguishing patients with SBIs from those with self-limited viral infections or non-infectious illnesses is often challenging due to non-specific clinical findings and laboratory parameters [4]. This can lead to unnecessary hospitalizations and increased healthcare burden. Recent studies have demonstrated that rapid identification of pathogens using molecular testing can reduce unnecessary antibiotic administration, duration of hospitalization, and costs [5]. However, the current epidemiology of systemic viral infections and their predictive values for excluding SBIs in febrile children is unknown.

In this study, we utilized an experimental multiplexed viral PCR test to describe the epidemiology of seven viral bloodstream infections in children presenting to the ED with suspected systemic infection who had blood cultures obtained at seven U.S. sites. Our cohort broadly included children with fever with and without a source, and special populations including neonates and immunocompromised children. We then performed statistical comparisons to determine the associations between viral bloodstream detections, bacterial cultures, and other standard-of-care tests.

## Methods

### Subject enrollment

This study received Institutional Review Board (IRB) approval for each of the seven geographically distinct U.S. sites for enrollment and testing over a period of 17 months (March 2017 to July 2018). Children < 18 years of age were enrolled prospectively if they met the following inclusion criteria: (1) Presented to the ED with suspected systemic infection indicated by a clinician-ordered blood culture; (2) parent/guardian gave written informed consent (and subject gave assent if of sufficient age and maturity); (3) blood specimen was collected during the ED visit or within 12 h of being admitted from the ED; (4) blood specimen was at least 500 μL; and (5) specimen could be aliquoted and tested or frozen within 1 day while stored at 4 °C. All specimens were coded such that they were not individually identifiable and so that the results of the experimental PCR testing could not be used to inform patient care. The IRB at one site (Emory University, IRB00096348) provided waiver of informed consent for clinical investigations involving no more than minimal risk to human subjects (https://www.fda.gov/media/106597/download).

Once enrolled, chart review was performed to abstract subject data including age, sex, hospitalization status, date of specimen collection, ED discharge diagnoses, and standard-of-care laboratory test results, including blood, cerebrospinal fluid (CSF), and urine cultures. For this study, an SBI was defined as a bacterial bloodstream infection, meningitis, or a urinary tract infection (UTI). A bacterial bloodstream infection was defined as growth of a pathogenic organism in one or more blood cultures. We excluded detection of presumed contaminating organisms, including coagulase-negative staphylococcus (CoNS), viridans streptococcus (other than *Streptococcus anginosus* group), *Micrococcus* sp., and *Corynebacterium* sp. Although these organisms can represent true pathogens in immunocompromised hosts, none were isolated on more than one repeated culture, and thus they were considered unlikely to be true pathogens. Bacterial meningitis was defined as growth of a pathogenic organism from the cerebrospinal fluid. Urinary tract infection was defined as having growth of a single pathogen at ≥50,000 colony forming units (CFU/mL), or if multiple potential pathogenic organisms were present, growth of a predominant pathogen at ≥50,000 CFU/mL. We excluded cultures with other polymicrobial growth, growth < 50,000 CFU/mL, growth of non-bacterial organisms (e.g. *Candida albicans*), growth of organisms which were not identified to the genus level (e.g. Gram-positive cocci), and growth of presumed contaminating organisms, such as CoNS and *Bacillus* sp.

### Multiplexed PCR testing

Whole blood was aliquoted and analyzed using an experimental multiplexed viral PCR (emvPCR) test based on the BioFire® FilmArray® System. The BioFire System is a test platform that is able to detect multiple pathogens from a variety of specimen types using an automated sample purification, multiplex-nested PCR, and amplicon melt analysis approach [6].

This study represents an analysis of results obtained from a pilot evaluation of the emvPCR test to identify viruses from whole blood of children presenting to the ED with suspected systemic infections. Viral targets on the emvPCR included adenovirus, cytomegalovirus (CMV), enterovirus, human parechovirus, herpes simplex virus 1 (HSV-1), herpes simplex virus 2 (HSV-2), and parvovirus B19. These targets were chosen because they are either common or high-risk pathogens associated with viremia in infants or children [7–9]. The emvPCR test provided automated interpretation of results as either “Detected” or “Not Detected” for each target, but did not provide PCR cycle threshold (Ct) values. During the course of the 17-month pilot study, the emvPCR test underwent two modifications to the thermocycling parameters to increase the sensitivity of the viral assays, which is an inherent limitation of the study.

### Statistical analysis

Statistical analyses were performed using Microsoft Excel and GraphPad Prism version 8.0.2. Continuous variables were expressed as medians and interquartile ranges (IQRs). Categorical variables were expressed as the absolute number of subjects and relative percentages. Comparisons were made using Student’s t-tests or one-way analysis of variance (ANOVA) for continuous variables and Fisher’s Exact tests for categorical variables with results reported as odds ratios with 95% confidence intervals. *P*-values ≤0.05 were considered statistically significant.

## Results

### Summary of emvPCR test findings

**Fig. 1:**
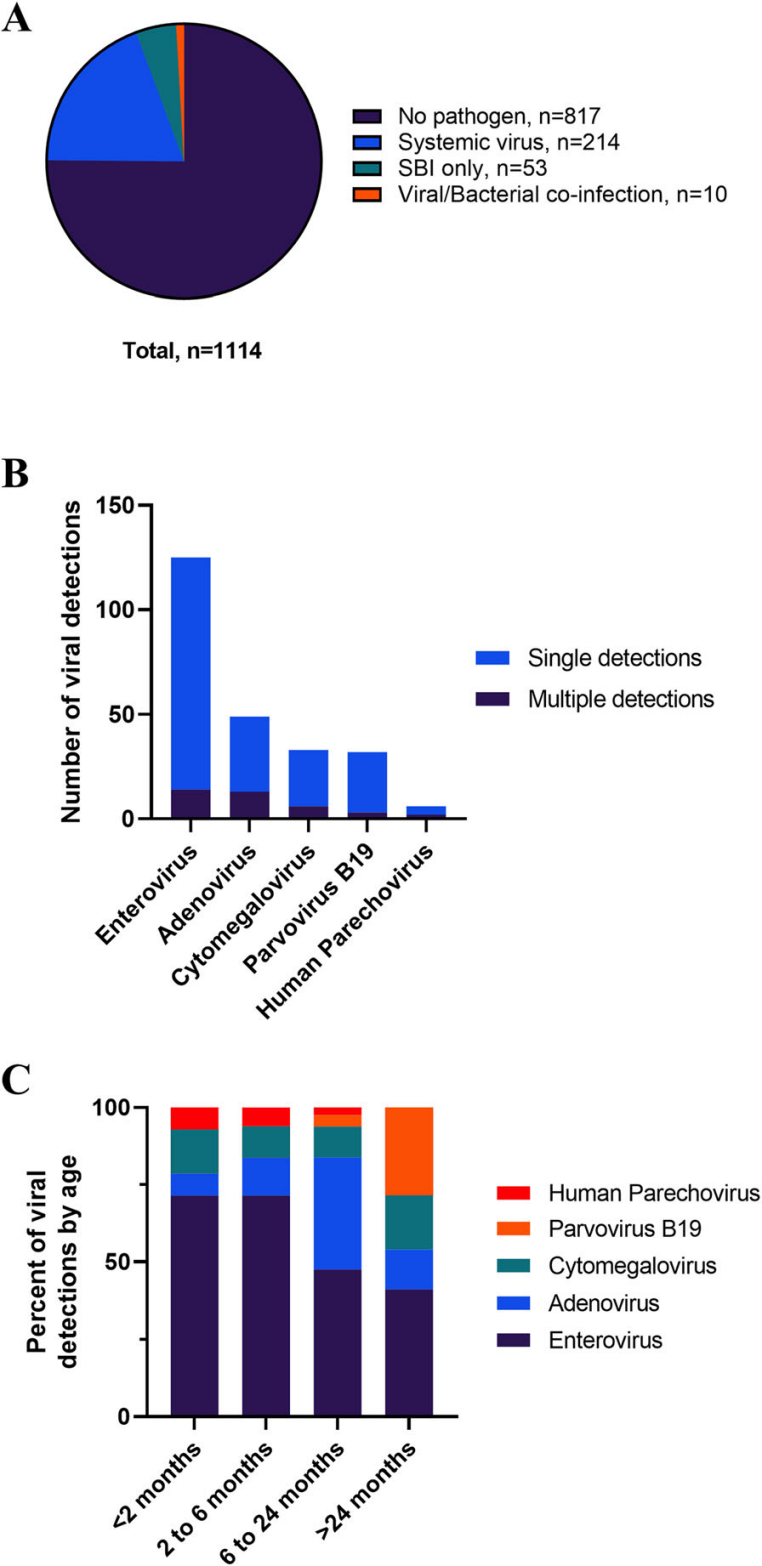
Viral detections in children presenting to the Emergency Department with suspected systemic infection. **a** Distribution of organisms detected in whole blood specimens by the emvPCR test or by standard-of-care bacterial cultures. **b** Viruses detected as single or co-infections. **c** Percentage of viruses detected by age group. emvPCR: experimental multiplexed viral PCR. SBI: Serious bacterial infection

Of the 1114 subjects enrolled at seven participating sites, 519 (46.6%) were female and the median age was 42 months (IQR 13 to 98.8 months). A total of 245 viruses were detected in whole blood collected from 224/1114 (20.1%) distinct patients (Fig. 1a), 10 (4.5%) of whom had concomitant SBIs. The most commonly detected virus was enterovirus (125/245, 51.0%), followed by adenovirus (49/245, 20.0%), CMV (33/245, 13.5%), parvovirus B19 (32/245, 13.1%), and human parechovirus (6/245, 2.5%). HSV-1 and HSV-2 were not detected. Of those who had viral detections, the majority were single viruses (Fig. 1b). However, 14 (6.3%) of subjects had two viruses detected, two (0.9%) had three viruses, and one patient (0.4%) had four viruses detected. Although enterovirus was the most frequently identified virus in all age groups (Fig. 1c), it accounted for a larger proportion of viral detections in the youngest age groups (ages < 2 months and 2 to 6 months) (*P* < 0.0001). In contrast, parvovirus B19 was only identified in children > 6 months of age.

### Summary of standard of care test results

Sixty-three subjects (63/1114, 5.7%) were found to have serious bacterial infections, which consisted of 29 bloodstream infections, 37 urinary tract infections, and one case of bacterial meningitis (Table 1). Four of these subjects had concurrent blood and urine cultures positive for the same organism.

**Table 1:** Standard-of-care (SOC) culture results with respect to viral pathogen detections by the emvPCR test

Viral targets on emvPCR test, n(%)	Bloodstream infection (***n*** = 29)	Urinary tract infection (***n*** = 37)^**a**^	Bacterial meningitis (***n*** = 1)^**b**^	Any SBI (***n*** = 63)^**c**^
**Adenovirus (*n*** **= 49)**	1/49 (2.0%)	1/18 (5.6%)	0/3 (0%)	1/49 (2.0%)
**Cytomegalovirus (*n*** **= 33)**	1/33 (3.0%)	1/13 (7.7%)	0/1 (0%)	2/33 (6.1%)
**Enterovirus (*n*** **= 125)**	0/125 (0%)	6/59 (10.2%)	0/13 (0%)	6/125 (4.8%)
**Human parechovirus (*n*** **= 6)**	1/6 (16.7%)	1/4 (25.0%)	0/2 (0%)	1/6 (16.7%)
**Parvovirus B19 (*n*** **= 32)**	0/32 (0%)	0/3 (0%)	0/0 (0%)	0/32 (0%)
**All viral positives (*n*** **= 224)**	3/224 (1.3%)	9/89 (10.1%)	0/21 (0%)	10/224 (4.5%)
**All viral negatives (*n*** **= 890)**	26/890 (2.9%)	28/294 (9.5%)	1/59 (1.7%)	53/890 (6.0%)
**Total**	29/1114 (2.6%)	37/383 (9.7%)	1/80 (1.3%)	63/1114 (5.7%)

^a^Denominator represents the number of subjects who had urine cultures performed

^b^Denominator represents the number of subjects who had CSF cultures performed

^c^Total number of SBIs may be less than sum of individual SBIs if individual subjects had multiple types of SBIs

Among the subjects with bacterial bloodstream infections, six had *Staphylococcus aureus* and five had *Escherichia coli* bacteremia. One subject each had *Salmonella Typhi, Samonella* non-*Typhi, Bacteroides fragilis*, *Enterococcus faecalis*, *S. anginosus* group, *Streptococcus agalactiae*, and *Streptococcus pneumoniae*. The remainder had Gram-negative rod bacteremia, with the exception of one subject who had both *Candida parapsilosis* and *Staphylococcus simulans* and was classified as having an SBI with diagnosis of a central-line associated tunnel infection. Thirty-five additional subjects had positive blood cultures growing presumed contaminants which were not isolated on subsequent blood cultures. These organisms were CoNS, viridans streptococcus (other than *S. anginosus* group), *Micrococcus* sp., and *Corynebacterium* sp. Thus, the majority of positive blood cultures in children presenting to the ED with suspected bloodstream infection (35/64, 54.7%) represented presumed contaminating organisms.

Of the 383 subjects who had urine cultures performed, 37 (9.7%) had a UTI defined as a urine culture growing a predominant uropathogen at ≥50,000 CFU/ mL. Thirty-four of these (34/37, 91.9%) screened positive by leukocyte esterase on urinalysis, whereas only 12 (12/37, 32.4%) had positive nitrites. All patients with UTIs with nitrites detected on their urinalyses were also positive for leukocyte esterase. An additional 38 (9.9%) of subjects grew contaminating organisms or pathogens at a concentration less than the defined threshold for the diagnosis of UTI. The single subject with bacterial meningitis grew *Neisseria meningitidis* from his CSF culture.

### Standard of care results with respect to viral detections

The frequency of SBIs in subjects with emvPCR viral detections was similar to those without viral infections (10/224 [4.5%] vs. 53/890 [6.0%], *P* = 0.5168) (Table 1). This was true for each type of SBI, including bacteremia (3/224 [1.3%] vs. 26/890 [2.9%], *P* = 0.2423), UTI (9/89 [10.1%] vs. 28/294 [9.5%], *P* = 0.8396), and meningitis (0/21 [0.0%] vs. 1/59 [1.7%], *P* > 0.9999). Among the 10 subjects who had viral and bacterial co-infections, all had single viral detections, the majority (6/10, 60%) had enterovirus and urinary tract infections, and 5/6 of these subjects were < 2 years of age. Only three subjects in our study had concurrent viral and bacterial bloodstream infections. One patient had *E. coli* urosepsis and adenovirus in the blood, the second had *E. coli* urosepsis and human parechovirus in the blood, and the third had *E. coli* sepsis and CMV detected in the blood. Thus, the negative predictive value of a viral bloodstream detection for bacteremia or bacterial meningitis was high (221/224, NPV = 98.7%). The sensitivity of a negative bloodstream PCR test for identifying SBI was (26/29) 90.0%, and the specificity was low (221/1085, 20.3%). The frequency of UTIs among participants with viral detections also remained appreciable (9/89, 10.1%) (NPV = 89.9%). The sensitivity of a negative bloodstream PCR test for identifying UTI was (28/9) 75.7%, while the specificity was low (80/346, 23.1%).

Participants with systemic viral infections alone were younger (Median 19 mos, IQR 7–56.6) than those with no pathogen detected (Median 48, IQR 17–111) (*P* < 0.0001), but similar in age to those SBI (Median 30 mos, IQR 9.5–86) (Table 2). Whereas younger age was associated with systemic viral infection, female sex was associated with lower odds of systemic viral infection compared to SBI (OR 0.46, 95% CI 0.25–0.82, *P* = 0.0094). Pyuria was also a significant predictor of UTI, but CSF pleocytosis (WBC > 5 cells/uL) was not a predictor of bacterial meningitis, although the frequency of bacterial meningitis was low, viral etiologies of meningoencephalitis were not analyzed, and we were unable to correct for confounding factors such as bloody lumbar punctures. The overall sensitivity and specificity of pyuria for predicting UTI among subjects who had urinalyses performed were (34/37) 91.9% and (516/586) 88.1% respectively. However, the sensitivity and specificity of leukocytosis (WBC ≥ 15,000 cells/uL) for any SBI was low at 47.6 and 78.9% respectively. Participants with systemic viral infections alone had decreased odds of hospitalization compared to children with SBIs (OR 0.22, 95% CI 0.11–0.44, *P* < 0.0001).

**Table 2:** Demographic and laboratory characteristics of subjects with systemic viral infections alone (by emvPCR test); any systemic bacterial infection (SBI); or no systemic virus or bacterial infection. *emvPCR* experimental multiplexed viral PCR, *WBC* white blood cell count, *LE* leukocyte esterase, *CSF* cerebrospinal fluid, *UTI* urinary tract infection

	Systemic virus only (***n*** = 214)	Any SBI (***n*** = 63)	No systemic virus or SBI (***n*** = 837)	Odds Ratio (95% CI)^**a**^	***P***-value^**a**^
Age, mos, median (IQ range)	**19 (7–56.5)**	**30 (9.5–86)**	**48 (17–111)**	**–**	0.2629 **< 0.0001**
Sex, female, n (%)	**91/214 (42.5%)**	**39/63 (61.9%)**	**389/837 (46.5%)**	**0.46 (0.25–0.82)** 0.85 (0.63–1.16)	**0.0094** 0.3180
Season, fall/winter, n (%)	144/214 (67.3%)	39/63 (61.9%)	532/837 (63.6%)	1.27 (0.71–2.22) 1.18 (0.86–1.62)	0.451 0.3376
Hospitalized, n (%)	**108/214 (50.5%)**	**52/63 (82.5%)**	469/837 (53.0%)	**0.22 (0.11–0.44)** 0.80 (0.60–1.07)	**< 0.0001** 0.1658
WBC, median (IQ range)	10.3 (7.3–15.0)	10.9 (8.6–18.1)	9.6 (6.5–13.6)	–	0.5399 0.2347
Pyuria (+LE), n (%)^b^	**11/207 (5.3%)**	**37/47 (78.7%)**	**56/366 (15.3%)**	**0.01 (0.01–0.04)** **0.31 (0.16–0.59)**	**< 0.0001** **0.0002**
CSF WBC > 5/μL, n (%)^c^	7/16 (43.8%)	4/11 (36.4%)	19/45 (42.2%)	1.36 (0.28–5.44) 1.06 (0.35–3.62)	> 0.9999 > 0.9999

^a^Top line compares “Systemic virus only” and “Any SBI” groups. Bottom line compares “Systemic virus only” and “No systemic virus or SBI” groups. For categorical variables, odds ratios and 95% confidence intervals were calculated using Fisher’s exact test. For continuous variables, *P*-values were calculated using one-way ANOVA with Tukey’s post-hoc multiple comparisons test

^b^Denominator represents the number of subjects who had urinalyses with leukocyte esterase performed

^c^Denominator represents the number of subjects who had CSF WBC performed

Although respiratory PCR diagnostics were not performed systematically on enrolled subjects, standard-of-care results for such tests were collected and recorded. Of the 1114 enrolled subjects, 438 (39.3%) had respiratory PCRs performed per standard of care on upper and/or lower respiratory specimens, and 217 (49.5%) had positive detections. The frequency of SBIs in subjects with respiratory viral infections was similar to those without respiratory viral infections (10/217 [4.6%] vs. 53/897 [5.9%], *P* = 0.5164) (Supplemental Table S1). This was true for each type of SBI, including bacteremia (4/218 [1.8%] vs. 25/897 [2.8%], *P* = 0.6340), UTI (30/293 [10.2%] vs. 37/383 [9.7%], *P* = 0.8342), and meningitis (0/20 [0.0%] vs. 1/60 [1.7%], *P* > 0.9999). Predictors of respiratory viral infections included younger age, male gender, fall/winter season, and lack of pyuria on screening urinalysis (Supplemental Table S2). The hospitalization rate for children with respiratory viral infections was lower than that of subjects with SBIs, but higher than that of subjects with no virus or SBI detected.

Of the 7 viruses included in the emvPCR panel, adenovirus and rhinovirus/enterovirus were also identified in respiratory PCR panels. Of the 29 adenovirus detections in the blood, 12 (41.4%) were also detected by respiratory PCR. Similarly, of the 49 patients who had enterovirus detections in the blood, 22 (44.9%) were also detected by respiratory PCR panel as rhinovirus/enterovirus. Concordance of results between whole blood and respiratory samples for both positive and negative detections for was 418 /438 (95.4%) for adenovirus and 344/438 (78.5%) for enterovirus. The lower concordance of rhinovirus/enterovirus PCR results may be attributable to the overrepresentation of rhinovirus compared to enterovirus in the nasopharynx, the lower likelihood of rhinovirus viremia, and the inability of the multiplexed PCR panels to distinguish between the two viruses.

## Discussion

In this study, we describe the epidemiology of seven viral bloodstream detections in children presenting to the ED with suspected systemic infection at seven geographically distinct U.S. sites. Viruses were detected in the bloodstream of 224/1114 (20.1%) subjects, the majority of which were enterovirus, followed by adenovirus, CMV, parvovirus B19, and human parechovirus. HSV-1 and HSV-2 were not detected. The predominance of enterovirus is consistent with previously reported data in young febrile children without a source [8, 10–12]. Of those who had positive viral detections, the majority were single viruses; however 17/224 (7.6%) of subjects had two or more viruses detected. While some of these were thought to represent true viral co-infections, CMV or parvovirus B19 co-detections may have alternatively represented viral latency or reactivation.

There was not a significant difference in the frequency of SBIs among subjects with viral bloodstream infections (10/224, 4.5%) vs. those without them (53/890, 6.0%), (*P* = 0.5168). These findings contrast with a recently published prospective single-center study by L’Huillier, et al. which found significant differences in the frequency of SBIs among virally infected and non-infected children < 3 years of age presenting to the ED with fever without a source. The discrepancy may in part be explained by the distinct patient population and by the higher frequency of SBIs observed among subjects without viral infections (20.5%) [10]. L’Huillier, et al. also included human herpesvirus 6 (HHV-6) in their study, which is a commonly reported cause of viremia in febrile young children. Similarly, Byington, *et. al* found significant differences in the frequency of SBIs among virally infected and non-infected infants < 90 days of age. The higher frequency of SBIs observed in this study may have been attributable to inclusion of pneumonia and soft tissue infections as SBIs, and may also be a reflection of the higher frequency of SBIs in young infants compared to the general pediatric population [9]. Nevertheless, both the L’Huillier study and our study found a similar risk of UTI in virally infected children. Of the ten subjects in our study with viral and bacterial co-infections, nine had UTIs, and six had concurrent enterovirus infections. This data corroborates multiple studies which have found the risk of UTI in febrile children with other clinically significant viral infections, including influenza [13] and respiratory syncytial virus (RSV) [14, 15] to be low, but still appreciable.

In the current era, UTIs are the most common form of SBI in febrile children without a source [2], which is consistent with the data reported herein. Although viral detections alone did not sufficiently exclude the diagnosis of UTI, the majority of patients with concurrent viral detections and UTIs had screening urinalyses positive for leukocyte esterase (8/9, 88.9%). The overall sensitivity and specificity of leukocyte esterase for UTI was moderately high, at 91.9 and 88.1%, respectively. Female sex was also a significant predictor of SBI, primarily due to a higher frequency of UTIs in females. Thus, the combination of viral molecular testing, clinical factors, and screening urinalyses were helpful predictors of SBIs. In contrast, leukocytosis was poorly predictive of viral or bacterial infection, which is consistent with previously reported literature [16]. CSF pleocytosis was similarly poorly predictive of SBI, although the frequency of bacterial meningitis in our study was very low and viral etiologies of meningoencephalitis were not analyzed.

Among febrile children without a clear diagnosis of SBI, Colvin, et al. found viral etiologies in up to 76% of subjects when both bloodstream and respiratory samples were systematically analyzed [8]. This estimate is higher than what was observed in the current study, likely because Colvin, et al. systematically analyzed respiratory samples for all patients and included additional viruses. Some of these viruses were of less certain clinical significance [HHV-6, bocavirus, WU virus, KI virus, BK virus], and a high rate of viruses were detected in subjects with SBIs and afebrile controls. Our study highlights that although viruses are frequently detected in the bloodstream of children presenting to the ED with suspected systemic infections, they are not adequate predictors of the presence or absence of SBIs alone. Although the detection of systemic viruses had high negative predictive value for bacteremia and meningitis, the risk of UTI remained appreciable. Thus, viral molecular testing of the bloodstream must be interpreted in the context of clinical risk factors and laboratory parameters, including screening urinalyses, markers of systemic inflammation, and clinical appearance of the child.

The interpretation of viral detections must also take into consideration virus- and host-specific factors, such as the timing and duration of viremia following infection and the age and immune status of the host. For example, in a prospective, multicenter study, Lafolie,et al. found that the sensitivity of blood enterovirus PCR testing is dependent upon the patient’s age and clinical presentation [17]. Detection of enterovirus was more sensitive in blood samples than CSF samples of newborns and infants with fever and sepsis-like diseases, but was less frequent in older children with suspected meningitis. In the case of adenovirus, viremia is much more common in immunocompromised children, who may experience prolonged viral shedding [18]. And in the case of parvovirus B19, acute viremia occurs 1–2 weeks after infection, but can persist at low levels for months to years [19]. Thus, detection of parvovirus B19 by DNA PCR in the bloodstream does not necessarily indicate acute infection.

This study had some key limitations. First, not all viruses of potential clinical importance were included in the multiplexed test. Omitted viruses included some herpesviruses such as Epstein-Barr virus (EBV), human herpesvirus 6 (HHV-6), and human herpesvirus 7 (HHV-7), whose results can be complicated by false positives in whole blood specimens due to latently infected B cells. This may have also been the case for CMV, another herpesvirus, which was included in the current study. Second, this study did not systematically assess for focal bacterial infections such as pneumonia or osteoarticular infections, as these are often clinical diagnoses that lack microbiologic confirmation. This may have led to an underestimation of the true frequency of SBI. Third, we identified only one case of bacterial meningitis, which limits our conclusions about the predictive value of viral detections on the diagnosis of meningitis. Fourth, two modifications to the emvPCR thermocycling conditions were implemented during the course of this study to improve assay performance characteristics. These iterations of development are inherent limitations of the study, and the true sensitivity and specificity of the experimental emvPCR compared to a gold standard are unknown. Finally, another inherent limitation is that this cohort study lacked a control population to ascertain frequencies of viral detections in afebrile children. This study was completed prior to the onset of the global pandemic of coronavirus disease 2019 (COVID-19) [20]. Thus, the impact of SARS-CoV-2 detection on patient management of children in the ED and the association of COVID-19 with SBIs remain important areas for future research.

## Conclusions

In conclusion, viral bloodstream detections were common in children presenting to the ED with suspected systemic infection who had blood culture obtained, and the predominant viruses were enterovirus and adenovirus. However, the frequency of SBIs among subjects with and without viral bloodstream detections were not significantly different. Viral bloodstream detections did have high negative predictive values for bacteremia and meningitis, but could not rule out UTI. Overall, these results indicate that viral molecular testing of the bloodstream may be useful for determining the likelihood of bacteremia or meningitis, but must be interpreted in the context of other clinical and laboratory parameters and the presence of risk factors for UTIs.

## Supplementary Information

**Additional file 1: Supplemental Table S1.** Demographic and laboratory characteristics of subjects with respiratory viral infections alone (by SOC PCR testing of upper or lower respiratory tract specimen); any systemic bacterial infection (SBI); or no respiratory virus or bacterial infection. SOC: Standard of Care. WBC: white blood cell count. LE: leukocyte esterase. CSF: cerebrospinal fluid. UTI: urinary tract infection. **Supplemental Table S2.** Standard-of-care (SOC) bacterial culture results with respect to respiratory viral pathogen detections by SOC PCR analysis of upper or lower respiratory tract specimens.

## Data Availability

The datasets used and/or analyzed during the current study are available from the corresponding author on reasonable request.

## Abbreviations

ANOVA: Analysis of variance
CFU: Colony forming unit
CMV: Cytomegalovirus
CoNS: Coagulase negative staphylococcus
COVID-19: Coronavirus disease 2019
CSF: Cerebrospinal fluid
Ct: Cycle threshold value
EBV: Epstein-Barr virus
ED: Emergency department
emvPCR: Experimental multiplexed viral polymerase chain reaction
HHV-6: Human herpesvirus 6
HHV-7: Human herpesvirus 7
HSV-1: Herpes simplex virus 1
HSV-2: Herpes simplex virus 2
IQR: Interquartile range
IRB: Institutional review board
NPV: Negative predictive value
OR: Odds ratio
PCR: Polymerase chain reaction
PPV: Positive predictive value
RSV: Respiratory syncytial virus
SARS-CoV-2: Severe acute respiratory syndrome associated coronavirus 2
SBI: Serious bacterial infection
UTI: Urinary tract infection
WBC: White blood cell
