# Supplementary material for: A multicenter evaluation of viral bloodstream detections in children presenting to the Emergency Department with suspected systemic infection
Source: BMC Pediatr. 2021 May 18;21:238. doi: 10.1186/s12887-021-02699-9 (PMC8129693; doi:10.1186/s12887-021-02699-9)
Supplement: Supplementary file 1 — Additional file 1: Supplemental Table S1. Demographic and laboratory characteristics of subjects with respiratory viral infections alone (by SOC PCR testing of upper or lower respiratory tract specimen); any systemic bacterial infection (SBI); or no respiratory virus or bacterial infection. SOC: Standard of Care. WBC: white blood cell count. LE: leukocyte esterase. CSF: cerebrospinal fluid. UTI: urinary tract infection. Supplemental Table S2. Standard-of-care (SOC) bacterial culture results with respect to respiratory viral pathogen detections by SOC PCR analysis of upper or lower respiratory tract specimens. [file 12887_2021_2699_MOESM1_ESM.docx]

**Supplemental Table S1.** Demographic and laboratory characteristics of subjects with respiratory viral infections alone (by SOC PCR testing of upper or lower respiratory tract specimen); any systemic bacterial infection (SBI); or no respiratory virus or bacterial infection. SOC: Standard of Care. WBC: white blood cell count. LE: leukocyte esterase. CSF: cerebrospinal fluid. UTI: urinary tract infection.

|  | **Bacterial Bloodstream infection (n=29)** | **Urinary tract infection (n=37)^a^** | **Bacterial meningitis (n=1)^b^** | **Any SBI (n=63)^c^** |
| --- | --- | --- | --- | --- |
|  |  |  |  |  |
| **All resp viral positives (n=217)** | 4/217 (1.8%) | 7/82 (8.5%) | 0/20 (0%) | 10/217 (4.6%) |
| **All resp viral negatives (n=897)** | 25/897 (2.8%) | 30/293 (10.2%) | 1/60 (1.7%) | 53/897 (5.9%) |
| **Total** | 29/1114 (2.6%) | 37/383 (9.7%) | 1/80 (1.3%) | 63/1114 (5.7%) |

^a^Denominator represents the number of subjects who had urine cultures performed.
^b^Denominator represents the number of subjects who had CSF cultures performed.
^c^Total number of SBIs may be less than sum of individual SBIs if individual subjects had multiple types of SBIs.

**Supplemental Table S2.** Standard-of-care (SOC) bacterial culture results with respect to respiratory viral pathogen detections by SOC PCR analysis of upper or lower respiratory tract specimens.

|  | **Respiratory virus only (n=207)** | **Any SBI (n=63)** | **No resp virus or SBI (n=844)** | **Odds Ratio (95% CI)^a^** | **P-value^a^** |
| --- | --- | --- | --- | --- | --- |
| Age, mos, median (IQ range) | 26 (11-60) | 30 (9.5-86) | 48 (15-111.2) | **---** | 0.4104  **<0.0001** |
| Sex, female, n (%) | 87/207 (42.0%) | 39/63 (61.9%) | 393/844 (46.6%) | **0.45 (0.25-0.81)**  0.83 (0.62-1.13) | **0.0063**  0.2441 |
| Season, fall/winter, n (%) | 154/207 (74.4%) | 39/63 (61.9%) | 522/844 (61.8%) | 1.79 (1.00-3.22)  1.79 (1.27-2.52) | 0.0580  0.0007 |
| Hospitalized, n (%) | 151/207 (72.9%) | 52/63 (82.5%) | 426/844 (50.5%) | 0.57 (0.27-1.16)  **2.65 (1.90-3.72)** | 0.1364  **<0.0001** |
| WBC, median (IQ range) | 9.4 (6.0-12.9) | 10.9 (8.6-18.1) | 9.8 (6.7-14.0) | **---** | 0.0531  0.3803 |
| Pyuria (+LE), n (%)^b^ | 6/102 (5.9%) | 37/47 (78.7%) | 61/376 (16.2%) | **0.02 (0.01-0.05)**  **0.32 (0.15-0.77)** | **<0.0001**  **0.0060** |
| CSF WBC >5/uL, n (%)^c^ | 5/15 (33.3%) | 4/11 (36.4%) | 21/46 (45.7%) | 0.88 (0.19-3.75)  0.60 (0.19-2.12) | >0.9999  0.5501 |

^a^Top line compares “Respiratory virus only” and “Any SBI” groups. Bottom line compares “Respiratory virus only” and “No respiratory virus or SBI” groups. For categorical variables, odds ratios and 95% confidence intervals were calculated using Fisher’s exact test. For continuous variables, P-values were calculated using one-way ANOVA with Tukey’s post-hoc multiple comparisons test.
^b^Denominator represents the number of subjects who had urinalyses with leukocyte esterase performed.
^c^Denominator represents the number of subjects who had CSF WBC performed.
